# How to predict the prognosis in juvenile-onset SLE?

**DOI:** 10.1016/j.ebiom.2021.103285

**Published:** 2021-03-25

**Authors:** Hiroyasu Yamamoto

**Affiliations:** Department of Biomedical Informatics, Division of Health Sciences, Osaka University Graduate School of Medicine, 1-7 Yamada-oka, Suita City 565-0871, Osaka, Japan

Systemic lupus erythematosus (SLE) is a heterogeneous chronic autoimmune inflammatory disorder that results from widespread immune complex deposition and secondary tissue injury, but its pathogenesis is complex [1]. Juvenile-onset SLE (JSLE), which develops before the age of 18 years and accounts for approximately 20% of all SLE cases, tends to have high disease activities with higher morbidity and mortality rates and require long-term immunosuppressive medications as compared with adult-onset SLE [2]. The main causes of death are renal dysfunction, malignant diseases, and cardiovascular disease (CVD). Owing to the recent improvement in its treatment, fulminant SLE incidence has been decreasing, and premature atherosclerosis greatly influences its mortality. However, to my knowledge, no guidelines have been established for monitoring or managing CVD in patients with SLE or JSLE. Patients at high risk of CVD must be identified, as not all patients with SLE have an equal risk of CVD.

Considering the future CVD risk in patients with JSLE and less traditional risk factors, clinicians should use a biomarker or biomarker panel that could easily identify patients. Some clinical studies reported several risk factors of CVD events, such as older age, male sex, dyslipidaemia, hypertension, renal disease, metabolic syndrome, disease duration, and high disease activity, which could be divided into traditional and SLE-specific factors [3]. In this issue of *EBioMedicine*, Robinson and co-workers identified a novel risk marker, apolipoprotein B (apoB)-to-apoA1 ratio, from an investigation integrating metabolomics, transcriptome, immune profiles, and clinical data of patients with JSLE. They verified its usefulness by conducting long-term observational clinical cohort studies and comparing clinical information between adult patients with SLE and mouse atherosclerotic models [4].

ApoB and apoA1 are major apolipoproteins involved in lipid transport and the pathogenic processes and complications of atherosclerosis. These are the major protein components in very-low-, intermediate-, and low-density lipoproteins, with one protein per particle, and in high-density lipoprotein particles, respectively [5]. Previous reports involving mostly adults demonstrated that the apoB:apoA1 ratio indicates the balance between atherogenic- and anti-atherogenic particles and the higher the value, the higher the risk of CVD. In this comprehensive analysis of >220 serum biomarkers in patients with JSLE who mostly had normal total cholesterol and triglyceride levels, apoB:apoA1 was also identified as a marker correlating with elevated metabolites in adult patients of SLE with atherosclerosis.

The high apoB:apoA1 ratio subgroup of patients with JSLE had a characteristic T-cell profile, together with the upregulated interferon (IFN) signalling pathway–related gene expression in the CD8^+^ T cells in the transcriptome analysis, consistent with the previous reports that peripheral blood mononuclear cells had a prominent expression of type I IFN-regulated genes in >95% of patients with JSLE. Such type I IFN signature is also found in the blood and tissues of patients with SLE and supposed to cause and exacerbate SLE, although the precise mechanisms are unknown [6,7]. Type I IFN activates the adaptive immune system and leads to inflammatory loops in which B cell-derived autoantibodies and/or immune complexes promote the production of type I IFN in plasmacytoid dendritic cells, resulting in sustained inflammation [8]. Therefore, the apoB:apoA1 ratio could be a useful marker not only for identifying the JSLE group at high risk of CVD but also for predicting future disease activity even in those with low disease activity. In fact, this marker positively correlated with disease activity during the following 3–7 years in this study.

The apoB:apoA1 ratio is one of the first identified biomarkers for predicting the prognosis of patients with JSLE, including the risk of CVD, but some issues remain in verifying its usefulness as an established marker in clinical practice. First, the age-related cut-off value may be considered because the apoB:apoA1 ratio varies with age. Second, this ratio should be carefully handled in some patients with JSLE because various autoantibodies could be produced in patients with autoimmune disease accompanied by autoimmune dyslipidaemia. Patients with JSLE with anti-apoC2 autoantibody-induced hypertriglyceridemia showed a disturbance in apolipoprotein fractions [9]. We could also assume that autoantibodies disrupt the measuring system of apolipoproteins even in the absence of overt autoimmune dyslipidaemia. Third, the combination with other markers would be a better biomarker panel. Other marker candidates might be found from the analysis of monocytes in which the tumour necrosis factor (TNF)-α expression, a main culprit of atherosclerosis, was upregulated in response to the epigenetic pattern changes [10]. Validation using a large number of patients with JSLE would provide more information on the apoB:apoA1 ratio and its related pathogenesis; it would lead to the discovery of a better marker and better lifelong health management of patients with JSLE.

## Declaration of Interests

No conflicts of interest to declare.
